# Expression of Human Endogenous Retrovirus *env* Genes in the Blood of Breast Cancer Patients

**DOI:** 10.3390/ijms15069173

**Published:** 2014-05-26

**Authors:** Dong-Won Rhyu, Yun-Jeong Kang, Mee-Sun Ock, Jung-Woo Eo, Yung-Hyun Choi, Wun-Jae Kim, Sun-Hee Leem, Joo-Mi Yi, Heui-Soo Kim, Hee-Jae Cha

**Affiliations:** 1 Department of Surgery, Kosin University College of Medicine, Busan 602-072, Korea; E-Mail: lovebreast@naver.com; 2 Department of Parasitology and Genetics, Kosin University College of Medicine, Busan 602-072, Korea; E-Mails: yunjung0531@hanmail.net (Y.-J.K.); sunnyock@kosin.ac.kr (M.-S.O.); 3 Department of Biological Sciences, College of Natural Sciences, Pusan National University, Busan 609-735, Korea; E-Mail: yjw1947@hanmail.net; 4 Department of Biochemistry, College of Oriental Medicine, Dongeui University, Busan 614-052, Korea; E-Mail: choiyh@deu.ac.kr; 5 Department of Urology, College of Medicine, Chungbuk National University, Cheongju 361-763, Korea; E-Mail: wjkim@chungbuk.ac.kr; 6 Department of Biological Science, Dong-A University, Busan 604-714, Korea; E-Mail: shleem@dau.ac.kr; 7 Dongnam Institute of Radiological & Medicine Sciences, Busan 619-953, Korea; E-Mail: jmyi@dirams.re.kr; 8 Institute for Medical Science, Kosin University College of Medicine, Busan 602-072, Korea

**Keywords:** HERV, *env*, mRNA, realtime PCR, blood, gene expression

## Abstract

Human endogenous retroviruses (HERV) *env* proteins have been recently reported to be significantly up-regulated in certain cancers. Specifically, mRNA and protein levels of HERV-K (HML-2) are up-regulated in the blood plasma or serum of breast cancer patients. Here, we collected blood samples of 49 breast cancer patients and analyzed mRNA expressions of various HERVs *env* genes including HERV-R, HERV-H, HERV-K, and HERV-P by real-time PCR. The expression of *env* genes were significantly increased in the blood of primary breast cancer patients but were decreased in patients undergoing chemotherapy to a similar level with benign patients. When we compared the group currently undergoing chemotherapy and those patients undergoing chemotherapy simultaneously with radiotherapy, HERVs *env* genes were reduced more in the chemotherapy only group, suggesting that chemotherapy is more effective in reducing HERV *env* gene expression than is radiotherapy. Among chemotherapy groups, HERV *env* gene expression was the lowest in the taxotere- or taxol-treated group, suggesting that taxotere and taxol can reduce HERVs *env* expression. These data suggest the potential to use HERVs *env* genes as a diagnosis marker for primary breast cancer, and further studies are needed to identify the mechanism and physiological significance of the reduction of HERV *env* gene expression during chemotherapy.

## 1. Introduction

Retroviruses, a form of mobile genetic elements, have important roles in both disease and primate evolution. Endogenous retroviruses (ERVs) was pathogenic, as well as being beneficial to a host in some cases. Furthermore, retroviruses also have played a key role in primate evolution that resulted in the incorporation of these elements into the human genome [1]. Human ERV (HERV) is now part of the human genome, occupying nearly 8% of the total genome, with approximately 98,000 ERV elements and fragments [2]. However, all HERVs appear to be defective due to major deletions or nonsense mutations, and no HERVs capable of replication had been identified, suggesting that most HERVs are merely traces of original viruses, having first integrated millions of years ago [3]. HERVs have been proposed to play a role in several forms of human cancer and autoimmune diseases, but conclusive evidence is still lacking [4,5,6]. Several reports have shown that HERVs are involved in several diseases including multiple sclerosis [7,8, ]schizophrenia [9], and HIV-infection [10].

Recent studies have suggested a strong relationship between HERVs and cancer based on the mRNA expression profile of HERVs in normal and cancer tissues [11,12,13,14,15,16]. One study compared the mRNA expression of HERVs in tumors to adjacent normal tissues and found high levels of HERV-K (HML-2) expression in testis tumor tissues, HERV-R (ERV3-1) in liver and lung tumor tissues, HERV-H in liver, lung, and testis tumor tissues, and HERV-P in colon and liver tumor tissues [16]. Another study analyzed multiple HERV-K (HML-2) surface envelope proteins in ovarian cancer using anti-HERV-K (HML-2)-specific antibody and found significantly increased expression in tumors with low malignant potential and low grade relative to expression in normal ovarian tissues. They also identified other classes of HERV *env* mRNAs, including HERV-R (ERV3-1) and HERV-E, which are expressed in these same ovarian cancer tissues. Anti-HERV antibodies including anti-ERV3 (30%), anti-HERV-E (40%) and anti-HERV-K (HML-2) (55%) were also detected in patients with ovarian cancer but not in normal female controls [17]. Furthermore, HERV-K (HML-2) mRNA and protein expressions in serum and blood were found to be significantly increased in cancer patients, and to be reduced by chemotherapy [18,19,20,21]. In addition, the HERV-K-MEL antigen, product of a pseudo-gene incorporated into the HERV-K *env* gene, was found to be significantly expressed in the majority of melanoma cells and other cancers [22,23].

We previously analyzed the HERV-R (ERV3-1) *env* protein in both adult human organs and in tumors using a tissue microarray and also compared the expression of HERV-R (ERV3-1) between normal and tumor tissues to study the relationship between HERV-R (ERV3-1) and tumor formation. HERV-R (ERV3-1) was highly expressed in normal organs and was significantly increased in various tumors, including lung adenocarcinoma, renal cell carcinoma, papillary carcinoma, hepatocellular carcinoma, and adenocarcinoma in the gastrointestinal tract [24]. Here, we analyzed and compared various HERV *env* gene mRNA expressions in the blood of 49 breast cancer patients to identify blood HERV *env* gene expression and to examine the potential use of HERVs as a breast cancer detection marker.

## 2. Results and Discussion

We collected blood samples of 10 healthy normal and 47 breast cancer patients containing benign tumors (Benign), primary breast cancers without chemotherapy (Primary no chemo), primary breast cancers undergoing chemotherapy (Primary chemo), and recurred and metastasized cancers. Recurred and metastasized cancers were divided into two groups: before chemotherapy (Meta prechemo) and undergoing chemotherapy (Meta chemo). However, Meta prechemo patients were treated with chemotherapy when they had primary tumors. The *env* genes expression of various HERVs including HERV-R (ERV3-1), HERV-H, HERV-K (HML-2), and HERV-P were analyzed by real-time PCR.

**Table 1:** Patients information and expressions of HERV *env* genes.

Patient No.	Gender	Age	Status	Stage	CTx	Rx	Expression (2^−ΔΔ*C*t^)			
							HERV-R	HERV-H	HERV-K	HERV-P
1	Female	25	Normal	NA	−	−	1.54806924	1.46936213	0.9275391	0.99967427
2	Female	31	Normal	NA	−	−	1.471890146	2.46791037	1.37724001	1.14023406
3	Male	25	Normal	NA	−	−	1.538228683	4.95879643	2.59526166	1.73282985
4	Male	38	Normal	NA	−	−	1.512746241	8.0583809	6.39294198	2.66203585
5	Female	25	Normal	NA	−	−	1.071157036	13.4086306	6.87891009	3.32217944
6	Male	31	Normal	NA	−	−	1.414870488	1.85316328	1.16342644	1.00558149
7	Female	25	Normal	NA	−	−	0.633655233	0.28319938	0.13652906	0.4591158
8	Male	26	Normal	NA	−	−	1.061327569	0.51662523	0.23777682	0.54624876
9	Male	25	Normal	NA	−	−	0.155951572	0.60970784	0.11957436	0.27466215
10	Male	26	Normal	NA	−	−	0.898300474	2.82527245	1.488831	0.80934684
11	Female	65	Benign	NA	−	−	0.969426438	6.60804418	1.16919319	2.01306906
12	Female	54	Benign	NA	−	−	1.316648275	10.5628214	2.48689685	3.75981932
13	Female	47	Benign	NA	−	−	1.131003453	7.46317751	2.59460214	1.52509241
14	Female	62	Benign	NA	−	−	1.235136082	11.1308356	2.20403429	1.42355345
15	Female	23	Benign	NA	−	−	3.067726784	5.04920645	0.92465046	2.9775588
16	Female	72	Benign	NA	−	−	1.958437833	3.11879775	0.72987096	0.95265781
17	Female	53	Benign	NA	−	−	8.088114008	0.20038179	0.03046887	9.30321907
18	Female	45	Primary	3c	−	−	5.319792596	18.2529364	6.3774514	8.69446145
19	Female	64	Primary	2a	−	−	16.14262545	39.6795383	10.1711671	15.4610172
20	Female	69	Primary	2a	−	−	9.608447428	8.99396124	4.93020169	8.97988312
21	Female	48	Primary	1	−	−	8.913363856	87.2262221	66.7816577	20.8662098
22	Female	57	Primary	1	−	−	3.813819767	18.7491939	9.5227615	7.94857735
23	Female	58	Primary	4	−	−	4.12807734	3.28552366	1.48206974	3.14537058
24	Female	75	Primary	2a	−	−	3.549551305	3.70504226	0.99168007	0.61249218
25	Female	49	Primary	0	−	−	6.627064679	33.2770737	63.1836773	16.2480724
26	Female	41	Primary	1	−	−	7.63980951	24.3444893	18.1808364	6.60914356
27	Female	64	Primary	2a	−	−	5.867326733	2.70049398	0.86049974	5.10306258
28	Female	44	Primary chemo	2b	FEC	+	0.583229932	3.42132858	1.35070567	0.70656621
29	Female	59	Primary chemo	1	FEC	−	1.922348628	19.9588497	13.4846546	2.12642504
30	Female	89	Primary chemo	2b	FEC	−	2.655413743	1.21267252	0.32439272	1.91732734
31	Female	78	Primary chemo	2a	CMF	+	6.824878361	9.60170093	4.13870667	4.4970014
32	Female	51	Primary chemo	1	FEC	+	1.27006588	6.65339183	1.08423689	7.41885563
33	Female	46	Primary chemo	1	FAC	−	1.703344207	28.0725848	5.5797997	7.00473271
34	Female	46	Primary chemo	3c	AC-taxol	+	0.730760217	2.65103674	0.43893524	1.69693347
35	Female	52	Primary chemo	2a	AC	+	3.813907886	22.6455656	5.9942531	6.20184155
36	Female	50	Primary chemo	1	FEC	+	2.83884353	14.931874	6.69126972	6.64835153
37	Female	61	Primary chemo	1	FEC	+	8.470829981	63.937481	24.7538325	21.6310231
38	Female	53	Primary chemo	1	CMF	+	2.113845596	12.3478675	3.84667871	3.80772688
39	Female	46	Primary chemo	2a	FEC	+	2.873687797	11.3739122	8.0083961	5.84659504
40	Female	48	Primary chemo	2a	FEC	+	2.776120133	25.2437908	17.7988649	10.6208697
41	Female	64	Primary chemo	2a	TA	+	0.544990631	4.74241966	1.46403157	1.24584967
42	Female	56	Primary chemo	1	FEC	+	3.620787059	16.8844506	13.754324	4.36202085
43	Female	53	Primary chemo	2a	CMF	−	1.035985157	2.41299234	1.01158204	1.64714017
44	Female	49	Primary chemo	2b	FEC	+	1.265233213	45.0438009	6.23611126	1.15212453
45	Female	36	Primary chemo	3b	TA	+	1.342234427	30.3275419	8.30499903	1.51382314
46	Female	50	Primary chemo	2b	CMF	+	3.154118071	95.4643386	29.5362207	2.34984131
47	Female	64	Primary chemo	1	FEC	+	2.795558755	66.8485023	27.5099193	4.24480025
48	Female	33	Meta no chemo	4	FEC	+	0.746425408	0.45963376	0.07435361	0.68133206
49	Female	47	Meta no chemo	4	CMF	+	3.49050715	24.6256517	3.02817098	2.55660628
50	Female	43	Meta no chemo	4	CMF	+	0.397355807	1.72938211	0.38039759	0.10406547
51	Female	49	Meta no chemo	4	CMF	+	9.724135523	10.9923149	2.68548004	6.7503548
52	Female	56	Meta no chemo	4	CMF	+	6.626911563	13.408011	7.89577019	3.87500096
53	Female	49	Meta chemo	4	CMF	+	1.383737227	0.53792987	0.12805599	1.64584674
54	Female	53	Meta chemo	4	FEC	+	2.838056544	0.50303244	0.26561588	0.84934936
55	Female	47	Meta chemo	4	AC	−	0.973399092	1.9376706	0.39927279	3.47905785
56	Female	59	Meta chemo	4	CMF	+	1.757514591	8.90854611	5.13375104	2.23178286
57	Female	60	Meta chemo	4	FEC	+	0.976259554	11.4398006	4.19425139	1.56341054

CTx: Chemotherapy; Rx: Radiotherapy; CMF: six cycles of cyclophsphamide, methotrexate, 5-FU; FEC: six cycles of 5-FU, epirubicin, cyclophosphamide; TA: six cycles of taxotere and adriamycin.

**Figure 1:**
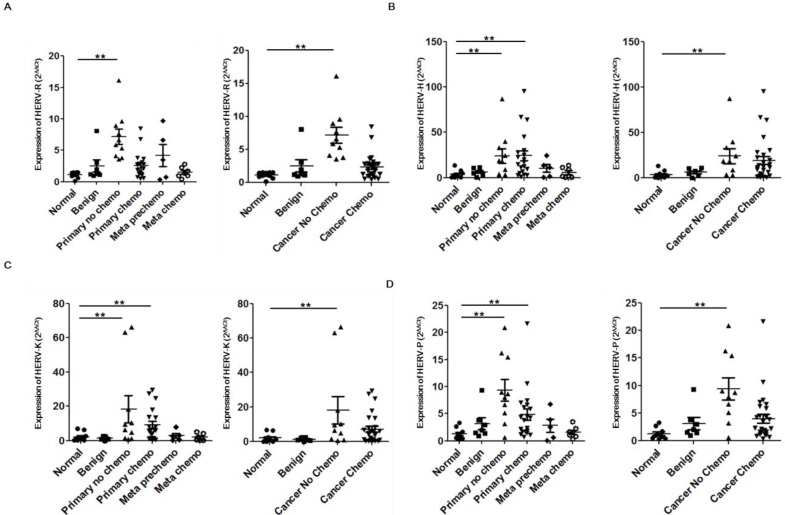
Expressions of HERVs *env* genes in the blood of breast cancer patients. Expression of the HERV-R (ERV3-1) (**A**); HERV-H (**B**); HERV-K (HML-2) (**C**); and HERV-P (**D**) *env* gene in the blood of breast cancer patients were analyzed by real-time PCR. Real-time PCR reactions were performed with blood total RNA of breast cancer patients including benign tumors (Benign), primary breast cancers without chemotherapy (Primary no chemo), primary breast cancers undergoing chemotherapy (Primary chemo), recurred and metastasized cancers before chemotherapy (Meta prechemo), and metastasized cancers undergoing chemotherapy (Meta chemo). Analyzed data were also compared to benign tumors (Benign), breast cancers without chemotherapy (Cancer No chemo), and breast cancers with chemotherapy (Cancer Chemo). Meta prechemo patients were ruled out in both Cancer No chemo and Cancer Chemo group because they are pre chemo stage but had experience of chemotherapy when they had primary cancer. Real-time PCR reactions were coupled to melting-curve analysis to confirm the amplification specificity. Non-template controls were included for each primer pair to check for any significant levels of contaminants. Real-time PCR was performed in three independent experiments with three different samples per group. The mRNA expression levels are presented relative to the control with calculated mean values and 95% confidence intervals. Statistical significance of differences between groups was determined using a two-tailed Student’s *t*-test. ** *p* < 0.01.

All patients’ information and expression score of each patient was described in Table 1. The expression of HERV-R (ERV3-1) *env* was significantly increased in the blood of Primary no chemo samples but reduced in patients undergoing or previously treated with chemotherapy to similar level with normal or benign patients (Figure 1A). HERV-H *env* expression was up-regulated in both the Primary no chemo and Primary chemo groups but was reduced in the Meta Prechemo and Meta chemo groups comparing with primary cancer patients. When we compared cancers having undergone chemotherapy and cancers not subjected to chemotherapy, we found that HERV-H *env* expression was also reduced in groups with chemotherapy (Figure 1B). The expression of HERV-K (HML-2) *env* in the Primary no chemo group was highly over-expressed (8.2-fold increase) compared to normal or benign group. HERV-K (HML-2) *env* expression was slightly reduced in the Primary chemo group but still showed a significant increase compared to normal or benign group. When we compared cancers with and without chemotherapy, we found that HERV-K (HML-2) expression, similar to HERV-R (ERV3-1), was reduced in groups with chemotherapy (Figure 1C). HERV-P *env* expression also showed a similar expression pattern to HERV-R (ERV3-1) *env*, including an increase in Primary no chemo group, and decrease in chemotherapy groups (Figure 1D). These data suggest that HERV *env* genes are significantly increased in the blood of primary breast tumor patients but this induction is reduced by chemotherapy.

**Figure 2:**
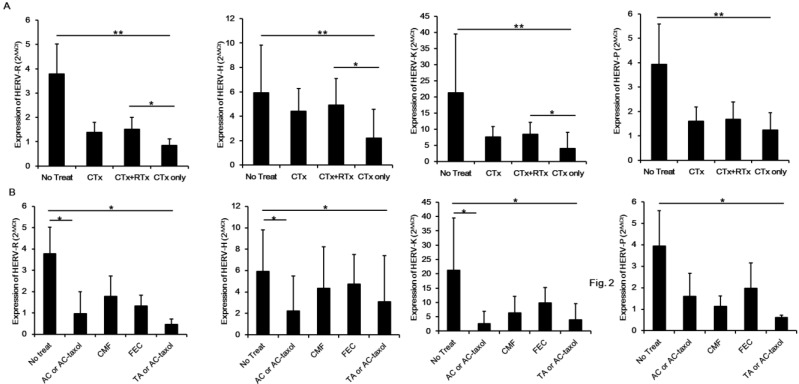
Analysis of HERV *env* genes expression according to chemotherapy protocol. Effect of chemotherapy and co-treated with radiotherapy on the reduction of HERV *env* genes in the blood of breast cancer patients (**A**); Expression of HERV *env* genes was compared by chemotherapy method including no chemotherapy (No Treat), chemotherapy included (CTx), chemotherapy accompanying radiotherapy (CTx+RTx), and chemotherapy only (CTx only) groups. Comparing the HERV *env* genes reduction according to chemotherapy protocol (**B**). The effect of each chemotherapy protocol on the reduction of HERVs *env* gene in the blood of breast cancer patients was analyzed. CMF: six cycles of cyclophsphamide, methotrexate, 5-FU; FEC: six cycles of 5-FU, epirubicin, cyclophosphamide; TA: six cycles of taxotere and adriamycin. * *p* < 0.05, ** *p* < 0.01.

In order to identify which treatment has a critical effect on reducing the expression of HERVs *env* genes, we analyzed data by classifying the patients into treatment groups. As shown in Figure 2A, the expression of HERV *env* genes was the lowest in the chemotherapy only group. Even the chemotherapy and radiotherapy group showed a higher level of HERV *env* gene expression. These data suggest that chemotherapy has a significant effect on reducing HERV *env* gene expression, and radiotherapy has no effect or may up-regulate the reduced expression of HERV *env* genes in chemotherapy-treated patients. We also analyzed the expression pattern of HERV *env* genes by classifying the chemotherapy methods. As shown in Figure 2B, taxotere- or taxol-containing therapy has the maximum effect on reducing HERV *env* gene expression in blood of breast cancer patients. This finding suggests that taxotere or taxol may reduce the expression of HERV *env* genes, although further studies are needed to identify the action mechanism and physiological roles of taxol on the reduction of HERV *env* genes.

The relationship between HERV-derived protein and breast cancer was first identified through the finding of the expression of HERV-K-MEL *env* gene in various tumors including breast cancer [23]. In addition, very high titers of HERV-K (HML-2) RNA in the plasma of patients with lymphomas and breast cancer measured by either reverse transcriptase PCR or nucleic acid sequence-based amplification [18]. That study also reported that the mRNA expression of HERV-K (HML-2) decreased dramatically with cancer treatment, and the presence of HERV-K (HML-2) virus-like particles in the plasma of lymphoma patients suggests that elements of the endogenous retrovirus HERV-K (HML-2) can be found in the blood of modern-day humans with certain cancers [18]. Other studies have since been conducted that suggest that HERV-K (HML-2) *env* could be a novel candidate prognostic marker and immunotherapeutic target for breast cancer [19,20]. HERV-K (HML-2) reverse transcriptase also has been reported to be a breast cancer prognostic marker [25]. An RT-PCR approach demonstrated that the HERV-R (ERV3-1) *env* gene was expressed in several human tissues (brain, prostrate, testis, kidney, placenta, thymus, and uterus) and cancer cells (RT4, BT-474, MCF7, OVCAR-3, LOX-IMVI, and AZ521). In addition, our previous reports showed HERV-R (ERV3-1) was highly expressed in certain tumors [24]. In case of the HERV-H *env* gene in human tissues, the *env* fragments were detected in the mRNA of several tissues (placenta, skeletal muscle, spleen, and thymus) and various cancer cells (RT4, BT-474, HCT-116, TE-1, UO-31, Jurkat, HepG2, A549, MCF7, OVCAR-3, MIA-PaCa-2, PC3, LOX-IMVI, AZ521, 2F7, U-937, and C-33A) by RT-PCR analysis [26]. Transcripts of HERV-P structural genes have also be detected in various human tissues and cancer cells, suggesting a potential role in carcinogenesis [27]. Quantitative real-time RT-PCR analysis between the cancer tissues and adjacent normal tissues in *env* genes of HERV-R, HERV-H, HERV-K, and HERV-P indicated that high levels of expression were detected in various cancer tissues compared to the adjacent normal tissues [16]. A recent study also confirmed the potential of HERV-K (HML-2) as a diagnostic marker for detecting early-stage breast cancer by detecting HERV-K (HML-2) protein and mRNA in the serum of breast cancer patient through ELISA and real-time PCR analysis, respectively [21].

Here, we first analyzed various HERV *env* mRNAs including HERV-R (ERV3-1), HERV-H, HERV-K (HML-2), and HERV-P in whole blood samples from breast cancer patients and compared the mRNA expression patterns of each HERV *env* gene. The expressions of HERV-K (HML-2) and other HERV *env* genes were significantly increased in the blood of breast cancer patients and decreased in the patient groups undergoing chemotherapy. These data agree with previous studies and also suggest other HERV candidates as diagnostic markers or therapeutic targets for breast cancer.

We also analyzed the patterns of HERV *env* gene reduction by chemotherapy. Among chemotherapy groups including chemotherapy and radiotherapy group and a chemotherapy only group, the expressions of all HERV *env* genes were the lowest in the chemotherapy only group, indicating that radiotherapy has no effect or increases the expression of HERV *env* genes. This data agrees with our previous report showing that radiation induces HERV-R (ERV3-1) *env* expression by epigenetic modulation [28]. Among the chemotherapy methods, we found that taxotere- or taxol-containing therapy has the maximum effect on reducing expression of all HERV *env* genes. Contreras-Galindo *et al.* found virus-like particles in the plasma of lymphoma patients [18] and suggested HERV-K *env* expression comes from these viral particles. However, it is also possible that this expression comes from the contamination of debris nucleic acids from lymphocytes, circulating tumor cells, or exosomes. In this study, we collected whole blood to include whole possibilities including virus, lymphocytes, circulating tumor cells, or exosomes but excluded the possibility of genomic DNA contamination by treating DNase before constructing cDNA from purified total RNA. It is also an important issue whether the decreased expression of HERV *env* genes correlates with remission or simply just the chemotherapy treatment. The Meta Pre Chemo and Meta Chemo group consisted mainly of patients having recurred breast cancers, which is much more malignant than primary tumor. These groups had treated or have been treating with chemotherapy and showed the significant low level of all types of HERV *env* genes comparing with No Chemo group, suggesting the possibility that chemotherapy reduces the expression of HER *env* genes (Figure 1).

It is possible that these viruses up-regulate the expression of HERV *env* genes in the blood of breast cancer patients but real viral particles were not yet defined. Circulating tumor cells also can be candidates up-regulating HERVs *env* levels in blood but still needs direct evidences to probe it. The mechanism of reduction of HERV *env* genes by chemotherapy is also unclear and requires further study.

## 3. Experimental Section

### 3.1. Blood Samples of Patients

Blood samples were collected from 10 normal and 47 breast cancer patients including 7 benign tumors (Benign), 10 primary breast cancers without chemotherapy (Primary no chemo), 20 primary breast cancers undergoing chemotherapy (Primary chemo), 5 recurred and metastasized cancers before chemotherapy (Meta prechemo), and 5 metastasized cancers undergoing chemotherapy (Meta chemo). All primary and metastasized tumors were invasive ductal carcinoma. Both the collection and analysis of all samples were approved by the Institutional Review Board of Kosin University Gospel Hospital. Informed consent was obtained from each patient enrolled in the study (IRB approval number: KUCM IRB 12-003).

### 3.2. Real-Time PCR Analysis

We purified total RNA from whole blood which contains all possible HERV *env* gene RNA from particles in plasma or serum, lymphocytes, and circulating tumors. We treated blood samples with RBC lysis buffer (BioLegend, San Diego, CA, USA) to remove red blood cells and added Trizol reagent (Invitrogen, Carlsbad, CA, USA) to purify total RNA according to the manufacturer’s guidelines. All purified RNA from all samples was treated with DNase before constructing cDNA to remove genomic DNA contamination. One microgram of total RNA was subjected to reverse transcription using the AffinityScrip™ Multiple Temperature cDNA synthesis kit (Stratagene, La Jolla, CA, USA). Relative quantitative real-time PCR was performed in a Applied Biosystems 7500 Fast Real-Time PCR System (Applied Biosystems, Carlsbad, CA, USA) using the SYBR^®^ Green PCR Master Mix (Applied Biosystems, Carlsbad, CA, USA) according to the manufacturer’s instructions with the primer sets we used in previous study [16] and described in Table 2. All samples were performed in duplicate and related to the expression of an appropriate housekeeping gene (GAPDH), as confirmed in previous work [16].

**Table 2:** Primers of HERVs *env* genes for realtime PCR analysis.

Target Gene	Primers		GenBank Accession No.
	Forward	Reverse	
HERV-R *env*	5'-CATGGGAAGCAAGGGAACT-3'	5'-CTTTCCCCAGCGAGCAATAC-3'	AC073210 from Chr.7q11.21
HERV-H *env*	5'-TTCACTCCATCCTTGGCTAT-3'	5'-CGTCGAGTATCTACGAGCAAT-3'	AJ289711 from Chr.2q24.3
HERV-K *env*	5'-CACAACTAAAGAAGCTGACG-3'	5'-CATAGGCCCAGTTGGTATAG-3'	AC074261 from Chr12q14.1
HERV-P *env*	5'-CAAGATTGGGTCCCCTCAC-3'	5'-CCTATGGGGTCTTTCCCTC-3'	DQ247958 from Chr.14q32.12

All PCR reactions were coupled to melting-curve analysis to confirm the amplification specificity (Figure S1). Non-template controls were included for each primer pair to check for any significant levels of contaminants. Real-time PCR was performed in three independent. The mRNA expression levels of HERV *env* genes in breast cancer patients are presented relative to the benign patients group as control with the calculated mean values and 95% confidence intervals. Statistical significance of the differences between groups was determined using a two-tailed Student’s *t*-test. *p* values less than 0.05 were considered statistically significant.

## 4. Conclusions

In conclusion, various HERV genes including HERV-R (ERV3-1), HERV-H, and HERV-P *env* show the same pattern of regulation as HERV-K (HML-2) and may be potent diagnostic markers and therapeutic targets for breast cancer patients.

## Supplementary Files

Supplementary File 1 Supplementary Information (PDF, 489 KB)
