# Supplementary figures and images for: Expression of Human Endogenous Retrovirus env Genes in the Blood of Breast Cancer Patients
Source: Int J Mol Sci. 2014 May 26;15(6):9173–83. doi: 10.3390/ijms15069173 (PMC4100088; doi:10.3390/ijms15069173)

# Supplementary Information

Figure S1. Comparative melt curves of all amplified products.

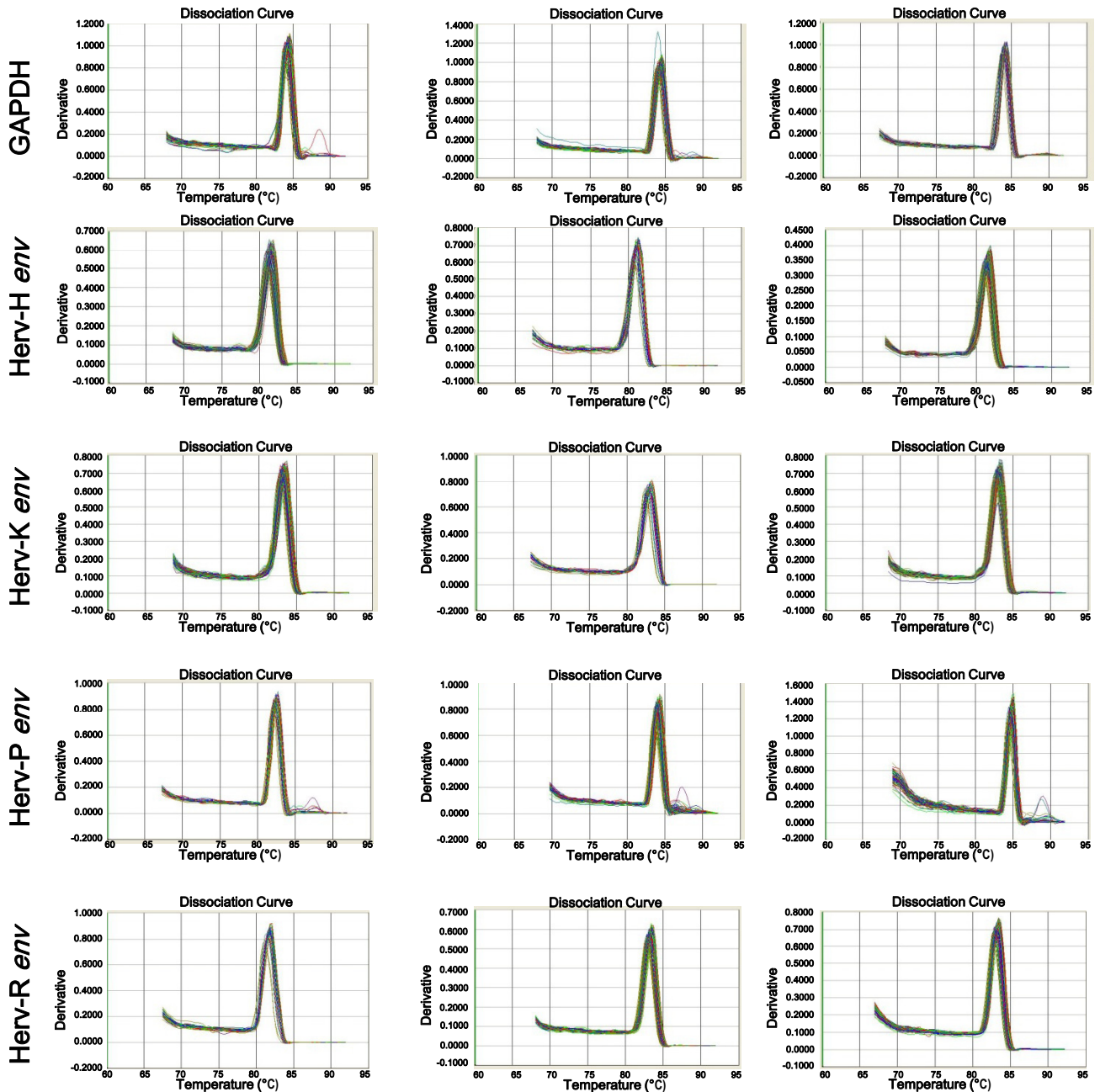

Supplement: Supplementary File 1 — Supplementary Information (PDF, 489 KB) [file ijms-15-09173-s001.pdf]
